# Identifying genetic networks underlying myometrial transition to labor

**DOI:** 10.1186/gb-2005-6-2-r12

**Published:** 2005-01-28

**Authors:** Nathan Salomonis, Nathalie Cotte, Alexander C Zambon, Katherine S Pollard, Karen Vranizan, Scott W Doniger, Gregory Dolganov, Bruce R Conklin

**Affiliations:** 1 Gladstone Institute of Cardiovascular Disease, 1650 Owens Street, San Francisco, CA 94158, USA; 2 Pharmaceutical Sciences and Pharmacogenomics Graduate Program, University of California, 513 Parnassus Avenue, San Francisco, CA 94143, USA; 3 Department of Medicine, Cardiovascular Research Institute, University of California, 505 Parnassus Avenue, San Francisco, CA 94143, USA; 4 Center for Biomolecular Science and Engineering, University of California, 1156 High Street, Santa Cruz, CA 95064, USA; 5 Functional Genomics Laboratory, University of California, Berkeley, CA 94720-3860, USA; 6 Cellular and Molecular Pharmacology, University of California, 600 16th Street, San Francisco, CA 94143-2140, USA

## Abstract

**Background:**

Early transition to labor remains a major cause of infant mortality, yet the causes are largely unknown. Although several marker genes have been identified, little is known about the underlying global gene expression patterns and pathways that orchestrate these striking changes.

**Results:**

We performed a detailed time-course study of over 9,000 genes in mouse myometrium at defined physiological states: non-pregnant, mid-gestation, late gestation, and postpartum. This dataset allowed us to identify distinct patterns of gene expression that correspond to phases of myometrial 'quiescence', 'term activation', and 'postpartum involution'. Using recently developed functional mapping tools (HOPACH (hierarchical ordered partitioning and collapsing hybrid) and GenMAPP 2.0), we have identified new potential transcriptional regulatory gene networks mediating the transition from quiescence to term activation.

**Conclusions:**

These results implicate the myometrium as an essential regulator of endocrine hormone (cortisol and progesterone synthesis) and signaling pathways (cyclic AMP and cyclic GMP stimulation) that direct quiescence via the transcripitional upregulation of both novel and previously associated regulators. With term activation, we observe the upregulation of cytoskeletal remodeling mediators (intermediate filaments), cell junctions, transcriptional regulators, and the coordinate downregulation of negative control checkpoints of smooth muscle contractile signaling. This analysis provides new evidence of multiple parallel mechanisms of uterine contractile regulation and presents new putative targets for regulating myometrial transformation and contraction.

## Abstract 2

A time course of gene expression at the onset of labor reveals transcriptional networks associated with activation of the uterine muscle and identifies targets for drugs to prevent premature labor.

## Background

The initiation of mammalian labor is a complex physiological process that requires the expression and secretion of many factors, both maternal and fetal [1,2]. The majority of these factors exert their effect on the myometrium, the smooth muscle responsible for expelling the fetus from the uterus. While species differences in labor regulation have been observed, several common signaling pathways and factors have been implicated as key regulators across species. During mid to late gestation, myometrial quiescence is maintained by several contractile inhibitors, such as relaxin, adrenomedullin, nitric oxide, prostacyclin and progesterone [1,2]. A number of these regulators stimulate cyclic AMP (cAMP)- and cGMP-mediated signaling pathways. Smooth muscle contraction is inhibited by the phosphorylation of myosin light-chain kinase by the cAMP-dependent protein kinase. This inhibition is believed to promote quiescence. In addition, the myometrium undergoes major structural changes throughout pregnancy that are required to generate the necessary contractile force for labor, including hypertrophy and hyperplasia of smooth muscle, connective tissue, focal adhesion, and cytoskeletal remodeling [3].

The transition to labor results in synchronous contractions of high amplitude and high frequency by the myometrium. Factors previously associated with the regulation of myometrial activation include the oxytocin receptor, gap junction protein connexin-43, voltage-gated calcium channels, prostaglandin receptor subtypes, estrogen, cortisol and transcription factors c-Jun and c-Fos. Most of these proteins participate in pathways that stimulate calcium release (for example, calcium-calmodulin G protein signaling) and the formation of intracellular junctions, leading to stimulation of contractions. Although several important components that regulate the initiation of labor have been identified, the mechanisms that guide this transition are poorly understood.

A difficult challenge in identifying the regulatory events that control the switch from myometrial quiescence to activation has been developing tools for examining whole-genome expression profiles in the context of known biology. Recent efforts to identify transcriptional changes from laboring and non-laboring human myometrium have proved valuable in identifying putative physiological regulators [4-8]; however, the lack of gestational time points examined has limited these approaches to interrogating only those genes with large fold-changes at term activation without exploring the global patterns of gene expression over the time-course of myometrial transformation. While gene profiling of the rodent uterus during gestation has proved fruitful in revealing some of the large-scale patterns of gene expression throughput pregnancy [5,9], there is still a critical need to improve the global view of myometrial gene expression with greater temporal resolution using newly developed bioinformatic tools.

To identify molecular mechanisms involved in the transition from myometrial quiescence to labor, we analyzed gene-expression changes in mouse myometrium at mid-gestation, throughout late gestation, and during the postpartum period. Our results reveal several novel patterns of expression occurring along the phases of myometrial quiescence to term activation and postpartum involution. Analysis of putative quiescence and term activation regulators in the context of well defined biological pathways revealed new putative functional roles for several previously unassociated genes in the suppression of contraction throughout gestation and activation of phase-dependent contractions at labor. This analysis further implicates the regulation of several novel pathways, including smooth muscle-extracellular matrix interactions throughout late gestation and cell junction-cytoskeletal interactions immediately before the onset of labor.

## Results

### Clustering of expression changes in gestational myometrium

Messenger RNA transcript levels were measured from isolated myometrium of 35 time-mated mice at four time-points of late gestation (14.5-18.5 days), at postpartum (6 and 24 hours after labor), and from a non-pregnant control group. In all, approximately 13,000 probe sets corresponding to around 9,000 unique cDNAs and expressed sequence tags (ESTs) were probed with oligonucleotide microarrays. About 35% of these transcripts were regulated throughout gestation and postpartum (14.5 days through 24 hours postpartum) using the criteria of *p *< 0.05 and a change in level of expression of more than 20% (fold-change 0.2).

**Figure 1:**
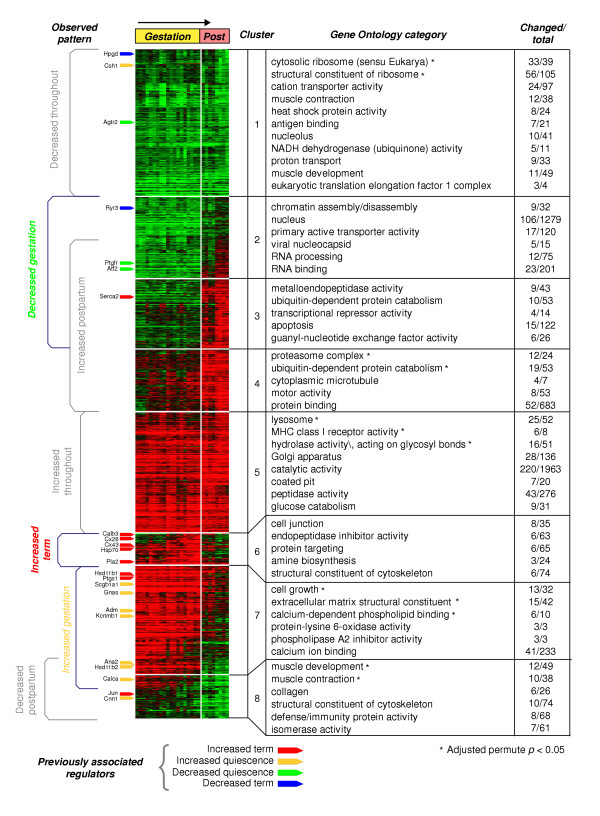
Clustering of myometrial expression profiles with HOPACH. Gene-expression profiles for 27 microarrays (vertical axis) and 4,510 probe sets (horizontal axis) are shown in the context of the HOPACH cluster map (non-pregnant data excluded). The array groups correspond to mid to late gestation (14.5, 16.5, 17.5 and 18.5 days) and postpartum (6 and 24 h). Eight clusters of genes are arranged vertically. Physiological phase groups are assigned on the basis of visual observation and association with previously associated regulators. MAPPFinder results are shown for the top-ranking distinct biological process, molecular function and cellular component groups based on a permuted *p*-value. Previously associated regulators of uterine quiescence and activation are indicated by a colored line next to the location of the corresponding gene probe set in the cluster map.

Analysis of these probe sets with HOPACH [10-12] revealed eight primary cluster groups and 133 subclusters. The majority of these clusters showed a clear association with known physiological phases of uterine gestation: quiescence (clusters 2, 3, 7 and 8), term activation (cluster 6), and postpartum involution (clusters 3, 4 and 7). In addition to these clusters, we observed two cluster groups with genes downregulated or upregulated throughout the analyzed time-course (clusters 1 and 5) (Figure 1).

### MAPPFinder analysis

To characterize the major biological processes, molecular functions, and cellular components associated with the HOPACH pattern groups, we used MAPPFinder (a component of GenMAPP version 2.0) [13-16]. MAPPFinder produced a statistically ranked list (based on *p*-value) of Gene Ontology (GO) biological categories associated with each cluster, from which the most significant nonsynonymous groups are listed (Figure 1, GO categories). In each cluster, several highly significant biological associations were identified (adjusted permutation *p *< 0.05).

### Association of expression clusters with previously associated uterine quiescence and activation genes

Gene expression groups associated with the maintenance of pregnancy (quiescence) or induction of labor (activation) were confirmed by mapping lists of previously associated regulators of uterine quiescence and activation onto our HOPACH cluster map. Extensive literature searches for such regulators identified 66 genes, of which 23 were regulated in our dataset (Figure 1, previously associated regulators). Genes hypothesized to regulate quiescence by transcriptional upregulation or secretion were largely associated with clusters 7 and 8 ('increased quiescence'), while putative activators of uterine activation were largely associated with cluster 6 ('increased term activation'). Although only three downregulated quiescence regulators were associated with HOPACH clusters, two of them mapped to cluster 2 ('decreased quiescence'), as predicted.

### Functional analysis of quiescence and term activation pattern groups

To further elucidate specific genes and pathways linked to the regulation of uterine quiescence and the initiation of labor, we examined pattern groups linked to quiescence and term activation, in the context of GO categories, GenMAPP pathway maps and literature associations. While low-magnitude fold-changes have been included within these functional analyses to broaden our survey of biological groups, we have largely restricted our discussion to transcripts with fold-changes greater than two.

### Upregulation of pathways of relaxation and remodeling during quiescence

**Figure 2:**
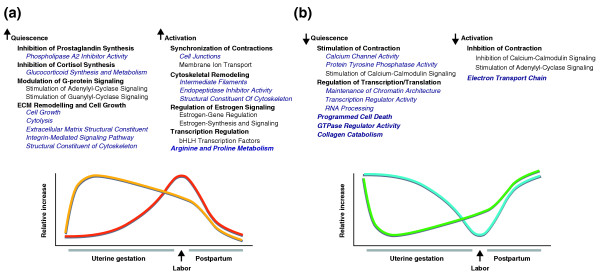
Association of quiescence and term activation pattern groups with biological pathways. Significant associations to GO classification groups and GenMAPP pathways were determined for each of the four expression pattern groups examined: Displayed are representative gene expression patterns for increased and decreased quiescence and term activation. **(a) **increased quiescence (yellow curve), increased activation (red curve); **(b) **decreased quiescence (green curve) and decreased activation (blue curve). GO terms and GenMAPP pathways highlighted by analysis with the program MAPPFinder are indicated by italicized blue text. Biological processes identified by literature association are indicated in black text. Parent biological categories are designated by bold text.

**Figure 3:**
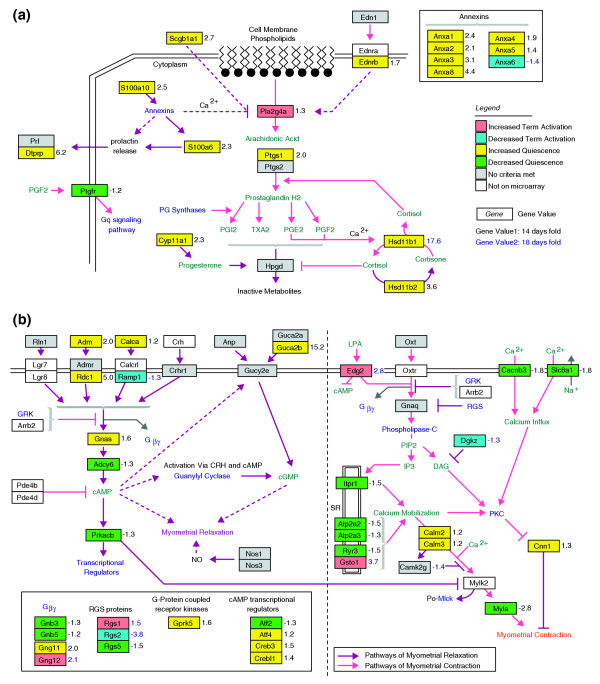
Analysis of pathways of uterine smooth muscle contraction. **(a) **Prostaglandin synthesis and **(b) **G-protein signaling pathways in the myometrium are overlaid with gene-expression color criterion and fold-changes from the program GenMAPP. Interactions suggested by results of this microarray analysis are included in these figures. Detailed gene-expression data, statistics and full gene annotations are available on the GenMAPP interactive version of these pathways online [40].

Analysis of genes upregulated throughout gestation (increased quiescence) revealed a number of biological categories associated with uterine quiescence. These categories contain a large number of highly regulated genes coupled to the inhibition of prostaglandin and cortisol synthesis, stimulation of cAMP and cGMP signaling pathways, extracellular matrix remodeling, cytolysis and regulation of cell growth (Figure 2, Table 1). To explore the potential relationships between the products of these transcriptionally regulated genes, we mapped the data onto respective metabolic and signaling pathways (Figure 3a,b).

Besides well established quiescence regulators (*Adm*, *Cgrp*, *Hsd11b2*, *Gnas*, *Cnn1 *and *Utg*; see Tables 1, 2, 3 for full gene names), several genes previously unassociated with the maintenance of quiescence were identified along the same or related biological pathways. The most highly regulated of these genes were those implicated in the induction of cGMP and cAMP signaling pathways (*Guca2b *and *Cmkor1*), genes for calcium-dependent phospholipid binding proteins (*Anxa1*, *Anxa2*, *Anxa3 *and *Anxa8*), and for the Anxa2 dimerization partner S100A10 (Figure 3a). Other changes in expression from this pattern group were observed among cytolysis-inducing proteases (granzymes B-G), regulators of cell growth (*Igfbp2 *and *Il1r2*), and transcriptional regulation (*Sfrp4 *and *Klf4*). Several of these and other genes were found to have highly reproducible patterns of expression using quantitative real-time PCR (TaqMan), with typically larger fold-changes produced by TaqMan than by GeneChip (consistent with the more conservative fold-changes typically produced after robust multi-array average (RMA) normalization) (see Additional data file 1).

Several genes for cAMP-response element transcription factors were also found within the increased quiescence group (*Atf4*, *Crebl1*, and *Creb3*, see Figure 3b). These are all members of a larger group of basic leucine zipper (bZip) transcription factors not previously associated with quiescence, which also includes the CCAAT/enhancer binding protein Cebpd, the Maf protein Mafk, the nuclear factor, interleukin-3, regulated Nfil3, and the X-box binding protein Xbp1, also upregulated with quiescence.

### Downregulation of mRNA processing and contraction-associated signaling during quiescence

MAPPFinder analysis of genes in the decreased quiescence group identified a wide variety of cell maintenance, transcription, and cell-signaling biological processes. Many of these GO categories were associated with the onset of labor (calcium-ion transport and protein tyrosine phosphatase activity) or myometrial postpartum involution (programmed cell death, collagen catabolism and ubiquitin-conjugating enzyme activity). These results are in accordance with the inhibition of contraction and suppression of cell death in late gestation. Unlike term-related biological processes, categories shared between the decreased quiescence and 'increased postpartum involution' group appear to be largely the result of a common transcript expression profile (Figure 1, cluster 3; Figure 2).

Although similar numbers of genes were downregulated or upregulated with quiescence (approximately 480-520 genes), very few genes were downregulated more than twofold at 14.5 days of gestation (Table 2). One of the most downregulated transcripts was the myosin light-chain gene *Myl4*; the Myl4 protein is the primary target for oxytocin-induced phosphorylation leading to uterine contraction at term. Several additional putative components of the oxytocin contractile signaling pathway (calcium-calmodulin signaling pathway) were also present in this expression group (Iptr1, Ryr3, Plcg1, and Atp2a2) (Figure 3b). Another large set of coordinately downregulated genes includes factors involved in RNA processing. Alternative splicing of putative quiescence and term activation regulators has been proposed to be a critical mechanism of the physiological switch to labor [17,18].

### Transition from remodeling and relaxation to cell-cell signaling and transcriptional regulation with activation of the myometrium at term

A large percentage of genes regulated with quiescence continued to be highly regulated at term. This result emphasizes the importance of expression changes immediately before labor to counteract the effects of quiescence. Consistent with the number of upregulated genes, MAPPFinder analysis of the increased term activation group identified a smaller set of GO terms and pathways. Prominent among these were genes associated with the formation of cell junctions, kinesin complexes and endopeptidase inhibitors. In addition, functionally related transcription factors (members of the basic helix-loop-helix (bHLH) family), ion transport proteins and ion transport regulators were coordinately upregulated at term.

Within these biological categories, several contractile regulators, both associated and unassociated with parturition, were highly upregulated. These genes include those for cell junction proteins (Cx43, Cx26, Ocln, and Dsp), the pulmonary smooth muscle contractile regulator and complement component C3, the estrogen signaling regulator Hsp70, the chloride conductance regulator Fxyd3 and the ryanodine receptor regulator Gsto1 (Table 3). These changes occurred in concert with the upregulation of signaling molecules, such as growth factors (Inhba, Inhbb), G-protein signaling components (Edg2, Gng12) (Figure 3b) and collagen catabolism proteins (Pep4, Mmp7). On the whole, however, this pattern group was dominated by the upregulation of genes encoding proteins that are largely epithelial-cell specific. Most prominent among these are the genes for the cytokeratin intermediate filament proteins, Krt2-7, Krt2-8, Krt1-18, and Krt1-19, and for the cytokeratin transcriptional regulator Elf3, which are among the most highly upregulated genes at term.

### Downregulation of pathways of calcium mobilization and G-protein signaling in term myometrium

HOPACH analysis with a metric that disregarded the direction of fold-change (see Additional data file 2) revealed a small number of downregulated genes at term that mirror the increased term activation group. Among these, we observed two highly downregulated genes: regulator of G-protein signaling 2 (*Rgs2*), a potent inactivator of Gαq-GTP bound activity, and inhibitor of DNA binding 2 (*Idb2*), a bHLH factor that heterodimerizes with other HLH proteins to inhibit their function. *Rgs2 *is one of the most downregulated genes throughout the gestation-postpartum time-course, in addition to being highly expressed in non-pregnant myometrium and throughout gestation. Additional term-downregulated G-protein signaling proteins that act to antagonize calcium-calmodulin signaling are illustrated in Figure 3b.

### Global mechanisms of transcriptional regulation

**Figure 4:**
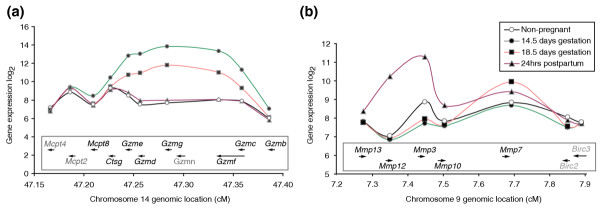
Association of genomic localization with co-regulation of expression. **(a,b) **Chromosomal gene clusters contain highly correlated expression changes among multiple members. Global patterns of gene expression within these genomic intervals are visualized by representing mean log expression for four of the myometrium time-point groups (non-pregnant, 14.5 and 18.5 days gestation, and 24 h postpartum), versus relative gene position on the chromosome. Gene strand orientation and position is designated by the orientation of arrows. Gene symbols above and below arrows are shown, where italicized black text indicates co-regulated genes (same HOPACH cluster) and italicized gray genes not co-regulated for (a) increased quiescence and (b) increased postpartum involution. Non-italicized gray text indicates genes not probed by the arrays.

One of the most prominent observations in this dataset is the highly significant correlation in the expression and genomic position of genes for eight serine-type endopeptidases (*Gzmb *through *Gzmg*, *Mcpt8 *and *Ctsg*) during the phase of quiescence. Genes within this multigene cluster undergo tight coordinate regulation in response to cell stimulation [19,20]. Examination of this expression cluster group in the context of genomic position reveals a novel pattern of positional gene regulation, where relative fold-change in expression increases from the peripheral members in the cluster to the center of the gene cluster (Figure 4a).

To determine whether other gene clusters exhibit a similar form of positional co-regulation, we developed a program to identify genomic intervals containing several coexpressed genes. Searching for regions with three or more members in a broad genomic interval (500 kilobases (kb)), we identified 11 clusters of genes that are co-localized and co-regulated (the same HOPACH cluster) [21]. Among these, we were able to identify at least one other gene cluster that possessed a genomic pattern of gene expression similar to that of the granzyme cluster, with genes maximally upregulated postpartum (Figure 4b). These genes, which encode several of the collagen catabolism matrix metalloproteinases, Mmp3, Mmp10, Mmp12 and Mmp13, are among the most highly upregulated genes postpartum. Because we do not have data from full genome arrays, it is difficult to determine if these co-regulated clusters of genes occur more frequently. However, these co-regulated gene clusters suggest coordinated gene regulation by an unknown mechanism.

## Discussion

This time-course analysis provides the first global view of gene-expression changes in mouse myometrium from uterine quiescence through the activation of the myometrium before labor and to its postpartum involution. Examination of multiple time points, the use of replicates, robust array normalization and powerful clustering tools enabled us to delineate and characterize unique patterns of gene expression throughout this physiological process. In addition to partitioning clusters of genes, analysis with the program HOPACH also provides us with a continuum of expression changes that reveals an overall transition in the expression of genes from one cluster group to another (Figure 1). Annotation of these clusters with GO terms provides a bird's eye view of the major processes regulating each of these pattern groups. These results support the hypothesis that mid-to-late gestation is dominated by changes in the expression of genes related to cell growth and extracellular-matrix remodeling (cluster 7), term gestation by changes in the content of cell junctions (cluster 6), and postpartum by targeted protein degradation, collagen digestion and apoptosis (clusters 3 and 4). Furthermore, results from genes upregulated throughout gestation and through postpartum suggest a continual local uterine immune response throughout this process (cluster 5). To help visualize the large-scale gene-expression changes in the context of myometrial physiology, we have depicted the data in an animation (see Additional data file 3) that summarizes our major findings.

A number of studies emphasize the importance of fetal regulation of the switch from quiescence to term activation, particularly increased cortisol and estrogen output from the fetal adrenal gland [1,2]. Interestingly, our studies provide evidence of a dynamic interplay between the myometrium and the fetus, particularly at the level of cortisol and progesterone synthesis (Figure 3a). Genes highly upregulated with quiescence include *Hsd11b*, which encodes an enzyme that converts cortisol to the inactive cortisone, and *Cyp11a1*, encoding an enzyme that promotes the synthesis of progesterone. Conversely, *Hsd11a*, coding for an enzyme that catalyzes the synthesis of cortisol, increased expression from 11- to 18-fold throughout gestation, suggesting that local regulation of cortisol levels are important for myometrial activation. While we observed the upregulation of the estrogen signaling regulator Hsp70, with term activation, downstream markers of estrogen action are among the most highly upregulated genes with term activation, supporting the role of the fetus in myometrial activation.

Examination of highly upregulated putative quiescence and term activation genes revealed several novel changes within important associated pathways for quiescence and activation (cAMP and cGMP signaling, calcium and calmodulin signaling and prostaglandin synthesis). Proteins encoded by these genes include Guca2b (uroguanylin), Anxa3, and Anxa8 with quiescence, and C3, Edg2, Gsto1 and Fxyd3 during activation (see Figure 3). These factors may represent novel targets for controlling the length of gestation. This is evidenced by the parallel observed upregulation of Guca2b from a recent microarray analysis of rat uterine gestation, where this factor has also been proposed to be a crucial regulator of cGMP-mediated smooth muscle relaxation throughout late pregnancy [9,22]. We have validated the expression patterns of a number of these genes using quantitative real-time PCR (see Additional data file 1). In addition to the candidates mentioned here, a number of other highly upregulated genes, whose functions have not been elucidated are also found in these two expression groups (see Additional data file 6).

Although a number of genes upregulated with quiescence or with term activation can be clearly implicated in the regulation of contractile pathways or uterine growth, several more groups of genes with little known functional connection to these processes were coordinately expressed. Highlighted among these groups are serine endopeptidases (granzymes) and bZip transcription factors, upregulated during quiescence, and endopeptidase inhibitors and bHLH factors, upregulated with term activation. In addition to its role in cytolysis, granzyme expression and secretion by T lymphocytes has been associated with the breakdown of extracellular matrix proteins in the uterus during pregnancy [18,23,24]. Interestingly, the upregulation of serine endopeptidases appears to be antagonized before the onset of labor by the upregulation of several serine endopeptidase inhibitors with term activation. A similar antagonistic relationship may also exist for bHLH factors upregulated at term with inhibitors of HLH function that are upregulated with quiescence and become downregulated at term.

Although the myometrium is considered to be relatively homogeneous, many of the largest changes in gene expression at term occurred in genes that are not normally associated with muscle, such as the keratins, tight junction and desmosome junction proteins. Indeed, altered gene expression due to changes in cell-type distribution or the invasion of the myometrium by the decidua and endometrium would not be distinguished if those changes occur consistently between gestational myometrium preparations. Further inspection of the literature reveals that the cytokeratins, which compose the bulk of this group, are expressed within smooth muscle and probably function as components of intermediate filaments of the cytoskeleton [25-28]. Furthermore, several components of desmosome spot junctions and hemidesmosomes, which interact with keratin intermediate filaments and the extracellular matrix to impart tensile strength between cells, are also upregulated with term activation (see Additional data file 3). These data suggest that an increase in rigidity-imparting cell junctions and remodeling of the cytoskeleton immediately before labor may promote coordinate contractions. However, further studies are needed to determine if cytokeratin expression at term occurs within resident or infiltrating cells.

In addition to the capability to group and annotate clusters of genes, pattern analysis with HOPACH can be used to interrogate gene clusters in the context of genomic location. For this analysis, we developed a program to isolate gene clusters that are likely to be co-regulated on the basis of genomic location, similar to other reported methods [29-32]. Using this program, we identified genomic regions that undergo correlated changes in gene expression associated with specific phases of the myometrial time-course. These groups highlight novel forms of gene regulation during quiescence and postpartum to coordinate cell responses (serine-protease activation and collagen catabolism). The prominent co-regulation among members of these two gene clusters further suggests that immune-cell trafficking and activation also play important roles in the progression towards labor and recovery from pregnancy.

## Conclusions

**Figure 5:**
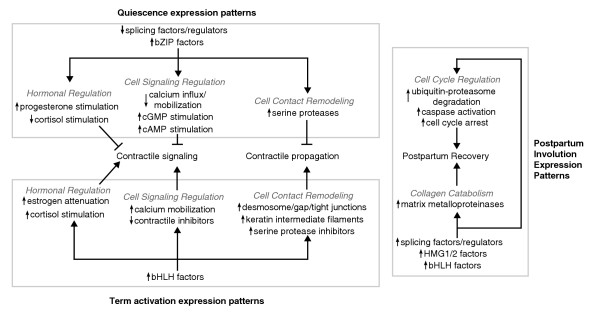
Proposed maternal model of uterine-directed contractile regulation. Theoretical model based on the major gene-expression pattern groups for quiescence, term activation and postpartum involution (light gray box outline). Arrows next to gene processes and functional groups indicate the predominant direction of fold-change as indicated by HOPACH analysis. This model proposes new roles for transcriptional regulators, regulators of mRNA processing, local hormone regulation, protease activity and cell junction formation in the control of both contractile signaling and contraction propagation in the myometrium during pregnancy. A model of postpartum involution is also presented, based on additional data (see Additional data files 4-6).

We have identified several highly regulated genes not previously associated with myometrial quiescence or activation, in addition to families of genes co-regulated at different phases of the myometrial time-course. In addition to providing new hypotheses about how the switch from quiescence to term activation may be facilitated (Figure 5), these data highlight several proteins that may serve as new candidate pharmacological targets for regulating myometrial contraction and thus the onset of labor. Such analyses will also be useful in predicting and correlating gene-expression changes in human pregnancy, where several time-points are often difficult to obtain [4-8]. Similar studies in other species using complementary methods of transcript measurement will also be necessary to validate these changes and understand the species-specific and regional myometrium transcriptional differences that probably occur. A detailed examination of the precise physiological roles of these regulators and mechanisms of regulation will be essential for developing a more detailed view of the regulation of labor.

## Materials and methods

### Tissue harvesting

FVB/N mice (Jackson Laboratory) were sacrificed in the morning (10 to noon) at 14.5 (*n *= 3), 16.5 (*n *= 4), 17.5 (*n *= 5), or 18.5 days (*n *= 7) after timed mating, and 6 (*n *= 4) or 24 h (*n *= 4) after delivery. Control myometrium was harvested from non-pregnant littermate females (*n *= 8) 1 day after timed mating with a vasectomized male. After dissection of both uterine horns, the tissue closest to the cervix was removed. Each horn was washed with PBS and opened longitudinally. Pups and placenta were discarded, and the decidua was removed by blunt dissection. The myometrium from each horn was then immediately frozen in liquid nitrogen and stored at -80°C.

### Sample preparation and microarray data normalization

For each sample, labeled cRNA was prepared from 20 μg purified total RNA and hybridized to Affymetrix Mu11k A and B arrays according to the manufacturer's instructions. Tissue from each mouse was hybridized individually to one array set. Microarrays were scanned at a photomultiplier tube (PMT) setting of 100%. Resulting .cel files were generated with Affymetrix Microarray Suite 5.0 and analyzed with RMA [33].

### Statistical analysis

To identify transcripts differing in mean expression across the seven experimental groups, *p*-values were calculated from a permutation test with the F-statistic function from the multtest package of Bioconductor [12,34]. Fold-changes in transcript levels were calculated from the mean log_2 _expression values of each time-point group versus the mean of non-pregnant controls. For cluster analysis, the dataset was filtered for probe sets with a *p *< 0.05 across the full expression time-course and a greater than 20% change in level of expression (positive or negative) for at least one time-point group versus non-pregnant controls. Additional filters were used downstream of clustering for genes related to uterine quiescence and term activation. For clusters related to quiescence and term activation, a change of more than 20% was required for the midgestation (14.5 days) and term (18.5 days) time points, respectively, versus non-pregnant controls.

### Clustering and pattern analysis

Gene expression clustering for 4,510 significant probe sets was performed using the program HOPACH (hierarchical ordered partitioning and collapsing hybrid), with uncentered correlation distance [10-12]. HOPACH produced a tree with six levels of clusters (eight primary level clusters and 133 main clusters). To examine expression patterns independently of the direction of the fold change, HOPACH was re-run with absolute uncentered correlation distance. Associations with GO biological process, molecular function, cellular component groups, and GenMAPP biological pathways were obtained with MAPPFinder 2.0, a part of the GenMAPP 2.0 application package [13-16]. A permuted *p*-value was calculated by MAPPFinder 2.0 to adjust for multiple hypothesis testing (see Additional data file 7). Because of the highly redundant nature of the oligonucleotide arrays used, redundant probe sets corresponding to a single gene were identified from the Affymetrix NetAFFX website [35].

### Real-time PCR validation of microarray data

Real-time reverse transcription PCR (RT-PCR) was used to validate the expression patterns of several highly regulated genes associated with specific phases of myometrium gestation. Gene-specific primers for multiplex real-time RT-PCR were designed for each gene of interest (*n *= 18) using Primer Express software (Perkin Elmer) and based on sequencing data from the National Center for Biotechnology Information (NCBI) databases and purchased from Biosearch Technologies. Sequence data for all oligos are available online [36]. Total RNA concentration and quality was assessed using the Agilent Bioanalyzer 2001. First-strand cDNA synthesis was performed using total cellular RNA (BD Biosciences Clontech), Powerscript reverse transcriptase (BD Biosciences Clontech), and random hexamer primers. Finally, an equivalent of 10 ng of total RNA from the first-strand cDNA synthesis reaction was used in 10 μl of each TaqMan gene quantification in 384-well format. Universal Master Mix for real-time PCR was purchased from Invitrogen Life Technologies. Raw data from an ABI Prism 7900 (Applied Biosystems) were processed into Excel spreadsheets and conversion of raw Ct values to relative gene copy numbers (GCN) was done as described previously [37]. Gene-expression analysis requires proper internal control genes for normalization. By using an endogenous control as an active reference, quantification of an mRNA target can be normalized for differences in the amount of total RNA added to each reaction. For this purpose, we used four mouse housekeeping genes - *PPIA*, *GAPDH*, *PGK1 *and *S9*. Moreover, using GeNorm [38], we selected *PGK1 *and *GAPDH *as the two most stable housekeeping genes across all 12 specimens and used their geometric means for normalization. Normalized data were graphed and compared to the data generated on similar specimens via microarrays. Genes could be broken down into the following groups: 13 genes with concordant microarray-TaqMan patterns; one false-negative result by microarray (*Acta2*); three genes with high TaqMan variability (*Mmp9*, *Krt19*, *Id1*); and one gene with evidence of alternative splicing (*Csb*) (see Additional data file 1). It should be noted that *Acta2 *baseline expression was relatively high for both microarray and TaqMan results. As both of these techniques probed different regions of the *Acta2 *gene, we cannot exclude the possibility of alternative splicing.

### Chromosomal localization analysis

We constructed a program to link HOPACH expression data to chromosome transcription start-site location and strand orientation, obtained from the Ensembl database [39]. Co-localized clusters of genes were identified as those genes clustered within a 500-kb genomic interval, belonging to the same HOPACH cluster, with a *z*-score >1.96, and an average pairwise Pearson correlation among cluster members of *r *>0.65 (see Additional data file 7 for calculation details and [21] for the full supplemental chromosome cluster lists).

## Additional data files

The following additional data are available with the online version of this article. Additional data file 1 is a figure showing the TaqMan vs GeneChip gene expression patterns. Relative fold changes (log base 2) are shown for 18 genes identified by these GeneChip studies to be differentially regulated throughout the myometrium gestation time-course. Combined standard errors are shown for each gestational time-point as compared to the non-pregnant control group. Additional data file 2 is a figure showing the HOPACH Absolute Value Pearson Correlation of Myometrial Expression Data. Gene expression data used for Pearson correlation HOPACH was used to generate a new set of clusters with a metric that disregards the direction of fold-change. Genes downregulated with term are identified based on association with genes upregulated at term from the non-absolute HOPACH analysis. Additional data file 3 is an animation of the summary and results, with a cartoon representation of myometrial transformation, general experimental design, results and conclusions. Additional data files 4 and 5 are Excel tables listing the MAPPFinder results. Nonsynonymous MAPPFinder GO categories for each expression pattern group are provided. Reanalysis with GenMAPP version 2.0 is required to visualize the genes that associate with each GO term. To download GenMAPP version 2.0, go to [16]. Additional data file 6 is a set of tables of cluster groups with annotations. Expression data, statistics, and biological groupings based on Gene Ontology annotations (via MAPPFinder analysis) and the literature are provided for 'Quiescence', 'Activation', and Postpartum 'Involution' gene lists. Additional data file 7 contains additional details of methods. Additional data file 8 contains the full expression dataset as an Excel file and Additional data file 9 is a GenMAPP format GEX file for use with GenMAPP format pathway maps (MAPP files). MAPP files can be downloaded from [16].

## Supplementary Material

Additional data file 1 A figure showing the TaqMan vs GeneChip gene expression patterns Click here for additional data file

Additional data file 2 A figure showing the HOPACH Absolute Value Pearson Correlation of Myometrial Expression Data Click here for additional data file

Additional data file 3 An animation of the summary and results, with a cartoon representation of myometrial transformation, general experimental design, results and conclusions Click here for additional data file

Additional data file 4 Excel tables listing the MAPPFinder results: HOPACH unique GO Click here for additional data file

Additional data file 5 Excel tables listing the MAPPFinder results: MAPPFinder quiescence, activation and postpartum Click here for additional data file

Additional data file 6 A set of tables of cluster groups with annotations Click here for additional data file

Additional data file 7 Additional details of methods Click here for additional data file

Additional data file 8 The full expression dataset as an Excel file Click here for additional data file

Additional data file 9 A GenMAPP format GEX file for use with GenMAPP format pathway maps (MAPP files) Click here for additional data file

## Figures and Tables

**Table 1:** Genes upregulated with quiescence

Increased gestation pattern group	Gene symbol	Fold-change at 14 days
		
**Prostaglandin and cortisol synthesis**		
Hydroxysteroid 11-beta dehydrogenase 1	*Hsd11b1*	10.6
Decidual/trophoblast prolactin-related protein	*Dtprp*	6.2
Hydroxysteroid 11-beta dehydrogenase 2	*Hsd11b2*	3.6
Cytochrome P450, 11a	*Cyp11a1*	2.3
Prostaglandin-endoperoxide synthase 1	*Ptgs1*	2.0
		
*Phospholipase inhibition*		
Annexin A8	*Anxa8*	4.4
Annexin A3	*Anxa3*	3.1
Uteroglobin	*Utg*	2.7
Calpactin	*S100a10*	2.5
Annexin A1	*Anxa1*	2.4
Annexin A2	*Anxa2*	2.1
		
**Proteolysis and peptidolysis**		
Kidney-derived aspartic protease-like protein	*Kdap*	8.2
CTLA-2-beta	*Ctla2b*	8.1
Cathepsin Z	*Ctsz*	3.1
Dipeptidase 1	*Dpep1*	3.1
Procollagen C-proteinase enhancer protein	*Pcolce*	2.6
Lipocalin 7	*Lcn7*	2.6
		
*Serine-type endopeptidases*		
Granzyme G	*Gzmg*	71.4
Granzyme D	*Gzmd*	45.7
Granzyme F	*Gzmf*	40.2
Granzyme E	*Gzme*	19.8
Granzyme C	*Gzmc*	10.7
RIKEN cDNA 2210021K23 gene	*2210021K23Rik*	2.9
Cathepsin G	*Ctsg*	2.2
Protease, serine, 11 (Igf binding)	*Prss11*	2.2
Granzyme B	*Gzmb*	2.1
		
*Protease inhibitors*		
Tissue factor pathway inhibitor 2	*Tfpi2*	4.2
Serine protease inhibitor 14	*Serpinb9e*	3.3
Plasma protease C1 inhibitor	*Serping1*	2.7
		
**Extracellular matrix remodeling and cell growth**		
*Regulation of cell growth*		
Insulin-like growth factor binding protein 2	*Igfbp2*	12.4
Interleukin 1 receptor, type II	*Il1r2*	5.0
Glucocorticoid-induced leucine zipper	*Gilz*	3.8
Tumor necrosis factor, alpha-induced protein 2	*Tnfaip2*	3.3
c-Fos induced growth factor	*Figf*	3.2
Related RAS viral (r-ras) oncogene homolog 2	*Rras2*	3.0
Cysteine rich protein 2	*Crip2*	2.9
MORF-related gene X	*Morf4l2*	2.6
Epithelial membrane protein 1	*Emp1*	2.5
Four and a half LIM domains 1	*Fhl1*	2.3
S100 calcium binding protein A6 (calcyclin)	*S100a6*	2.3
Insulin-like growth factor binding protein 6	*Igfbp6*	2.1
Transforming growth factor, beta 2	*Tgfb2*	2.0
		
*Integrin-mediated signaling pathway*		
Secreted phosphoprotein 1	*Spp1*	17.3
Connective tissue growth factor	*Ctgf*	2.8
Caveolin, caveolae protein	*Cav*	2.5
Ras homolog gene family, member A2	*Arha*	2.4
		
*Structural constituent of cytoskeleton*		
Gelsolin	*Gsn*	2.4
Tropomyosin 4	*Tpm4*	3.1
Tubulin, beta 2	*Tubb2*	2.2
		
*Extracellular matrix structural constituent*		
Microfibrillar associated protein 5	*Mfap5-pending*	6.9
Elastin	*Eln*	3.1
Procollagen, type XI, alpha 1	*Col11a1*	3.0
Fibromodulin	*Fmod*	2.4
Fibrillin 1	*Fbn1*	2.3
Procollagen, type V, alpha 2	*Col5a2*	2.2
Laminin, gamma 1	*Lamc1*	2.2
Procollagen, type I, alpha 2	*Col1a2*	2.2
		
**G-protein signaling**		
Guanylate cyclase activator 2b	*Guca2b*	15.2
Chemokine orphan receptor 1	*Cmkor1*	5.0
Adrenomedullin	*Adm*	2.0
Guanine nucleotide binding protein, gamma 11	*Gng11*	2.0
		
**Transcriptional regulation**		
Secreted frizzled-related sequence protein 4	*Sfrp4*	4.2
Kruppel-like factor 4	*Klf4*	3.0
C/EBP delta	*Cebpd*	2.3
Inhibitor of DNA binding 1	*Idb1*	2.1
X-box binding protein 1	*Xbp1*	2.0
Kruppel-like factor 2	*Klf2*	2.0

Only upregulated genes with a relative fold-change of 2 or more versus non-pregnant mice at 14.5 days gestation and linked to biological categories highlighted by the expression analysis are shown. Full gene lists can be obtained online (see Additional data file 6).

**Table 2:** Genes downregulated with quiescence

Decreased gestation pattern group	Gene symbol	Fold-change at 14 days
		
**Regulation of cell growth**		
Myosin light chain, alkali, cardiac atria	*Myl4*	-2.8
N-myc downstream regulated 2	*Ndr2*	-2.7
Actin, beta, cytoplasmic	*Actb*	-2.2
		
**Calmodulin signaling**		
MARCKS-like protein	*Mlp*	-2.2
		
**Proteolysis**		
Matrix metalloproteinase 3	*Mmp3*	-2.2
		
**Ion channels**		
Expressed sequence AW538430	*Kctd12*	-2.9
		
**Transcriptional regulation**		
SRY-box containing gene 4	*Sox4*	-2.9
Homeobox protein Meis2	*Mrg1*	-2.5
Special AT-rich sequence binding protein 1	*Satb1*	-2.1
D site albumin promoter binding protein	*Dbp*	-2.1
RIKEN cDNA 1110033A15 gene	*1110033A15Rik*	-2.1
Myeloid ecotropic viral integration site 1	*Meis1*	-2.0
		
**Regulation of alternative splicing**		
CDC-like kinase	*Clk*	-2.1

Only downregulated genes with a relative fold-change of 2 or more versus non-pregnant mice at 14.5 days gestation and linked to biological categories highlighted by the expression analysis are shown. Full gene lists can be obtained online (see Additional data file 6).

**Table 3:** Genes upregulated with term activation

	Gene symbol	Fold-change at 18 days
**Regulation of cell growth**		
Inhibin beta-B	*Inhbb*	3.1
Inhibin beta-A	*Inhba*	2.2
		
*Cell death*		
Growth arrest and DNA-damage-inducible 45 γ	*Gadd45g*	3.2
Baculoviral IAP repeat-containing 1a	*Birc1a*	2.1
Clusterin	*Clu*	2.0
		
**Cell junctions**		
Occludin	*Ocln*	2.8
Gap junction membrane channel protein α1	*Cx43*	2.8
Desmoplakin	*Dsp*	2.8
		
**G-protein signaling**		
Lysophosphatidic acid receptor Edg-2	*Edg2*	2.8
Guanine nucleotide binding protein, γ12	*Gng12*	2.1
		
**Structural constituent of cytoskeleton**		
Villin 2	*Vil2*	3.1
		
*Kinesin complex*		
Keratin complex 1, acidic, gene 19	*Krt1-19*	7.8
Keratin complex 2, basic, gene 7	*Krt2-7*	4.6
Keratin complex 2, basic, gene 8	*Krt2-8*	4.6
Keratin complex 1, acidic, gene 18	*Krt1-18*	4.5
Surfactant associated protein D	*Sftpd*	3.4
		
**Metabolism and biosynthetic reactions**		
Lipoprotein lipase	*Lpl*	4.5
Aldehyde dehydrogenase family 1, subfamily A2	*Aldh1a2*	3.9
Glutathione S-transferase omega 1	*Gsto1*	3.7
Branched chain aminotransferase 1, cytosolic	*Bcat1*	3.4
Protein phosphatase 1, regulatory subunit 3C	*Ppp1r3c*	2.2
Carbonic anhydrase 2	*Car2*	2.1
		
**Proteolysis and peptidolysis**		
Cytosolic nonspecific dipeptidase	*0610010E05Rik*	3.2
Transmembrane protease, serine 2	*Tmprss2*	2.1
Kallikrein 5	*Klk5*	2.1
		
*Collagen catabolism*		
Peptidase 4	*Pep4*	2.3
Matrix metalloproteinase 7	*Mmp7*	2.2
		
*Proteolysis inhibitors*		
Complement component 3	*C3*	4.3
RIKEN cDNA 1600023A02 gene	*1600023A02Rik*	2.9
Extracellular proteinase inhibitor	*Expi*	2.8
		
**Transcriptional regulation**		
*Transcription factors*		
Myeloblastosis oncogene	*Myb*	2.5
Hairy and enhancer of split 1	*Hes1*	2.3
E74-like factor 3	*Elf3*	2.1
		
*Androgen regulation*		
Kidney androgen regulated protein	*Kap*	33.9
Heat shock protein 4	*Hspa4*	3.1
Alpha fetoprotein	*Afp*	3.1
		
**Transport**		
FXYD domain-containing ion transport regulator 3	*Fxyd3*	2.8
Lipocalin 2	*Lcn2*	2.5
Lactotransferrin	*Ltf*	2.2
Solute carrier family 16, member 1	*Slc16a1*	2.1
Fatty acid binding protein 5, epidermal	*Fabp5*	2.0

Only upregulated genes with a relative fold-change of 2 or more versus non-pregnant mice at 18.5 days gestation and linked to biological categories highlighted by the expression analysis are shown. Full gene lists can be obtained online (see Additional data file 6).
