# Supplementary material for: Identifying genetic networks underlying myometrial transition to labor
Source: Genome Biol. 2005 Jan 28;6(2):R12. doi: 10.1186/gb-2005-6-2-r12 (PMC551532; doi:10.1186/gb-2005-6-2-r12)
Supplement: Additional data file 1 — A figure showing the TaqMan vs GeneChip gene expression patterns [file gb-2005-6-2-r12-s1.pdf]

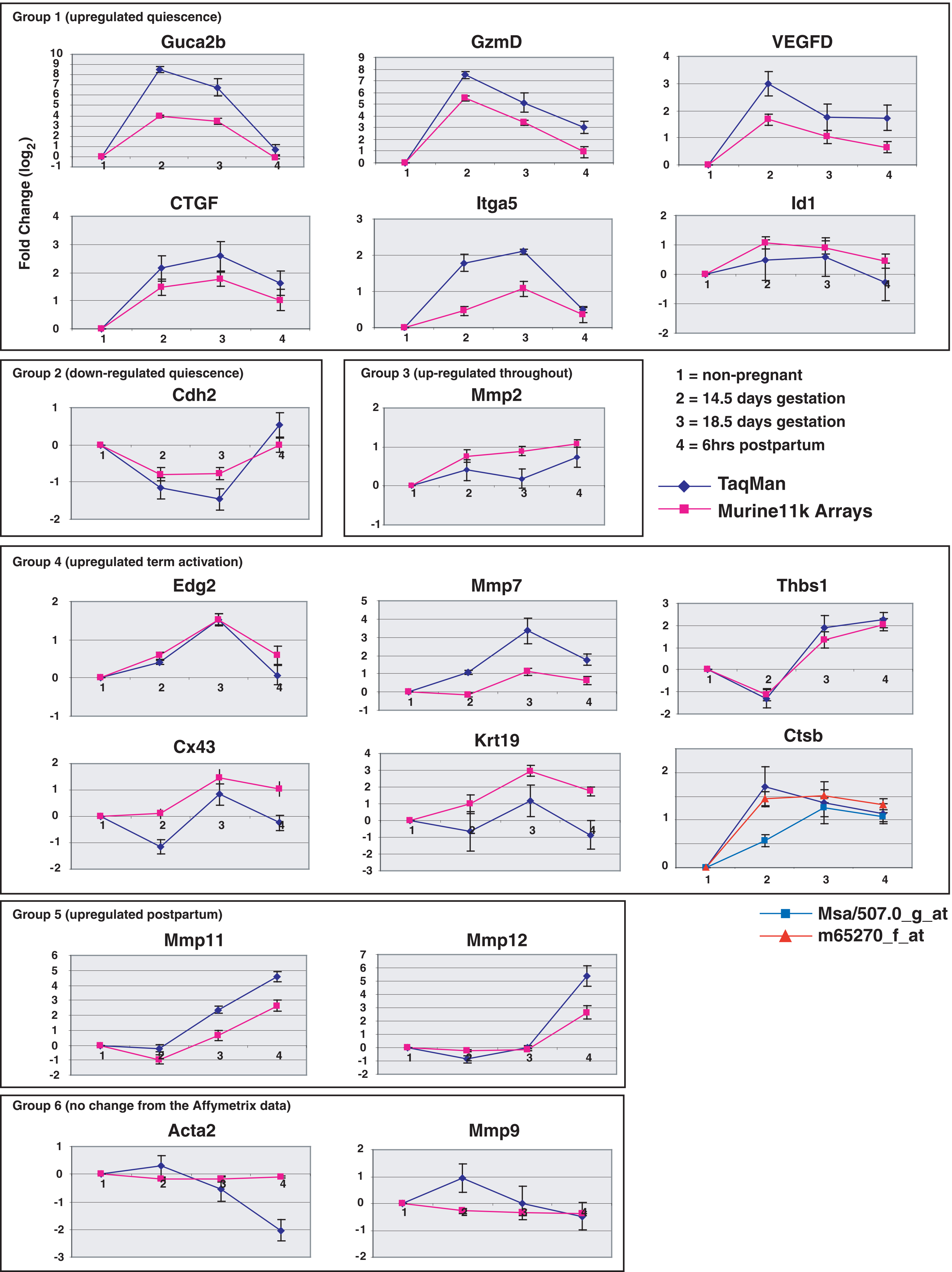
Group 1 (upregulated quiescence)
Guca2b
GzmD
VEGFD
CTGF
Itga5
Id1
Fold Change (log2)
Group 2 (down-regulated quiescence)
Cdh2
Group 3 (up-regulated throughout)
Mmp2
1 = non-pregnant
2 = 14.5 days gestation
3 = 18.5 days gestation
4 = 6hrs postpartum
TaqMan
Murine11k Arrays
Group 4 (upregulated term activation)
Edg2
Mmp7
Thbs1
Cx43
Krt19
Ctsb
Msa/507.0_g_at
m65270_f_at
Group 5 (upregulated postpartum)
Mmp11
Mmp12
Group 6 (no change from the Affymetrix data)
Acta2
Mmp9
