# Supplementary figures and images for: Identifying genetic networks underlying myometrial transition to labor
Source: Genome Biol. 2005 Jan 28;6(2):R12. doi: 10.1186/gb-2005-6-2-r12 (PMC551532; doi:10.1186/gb-2005-6-2-r12)

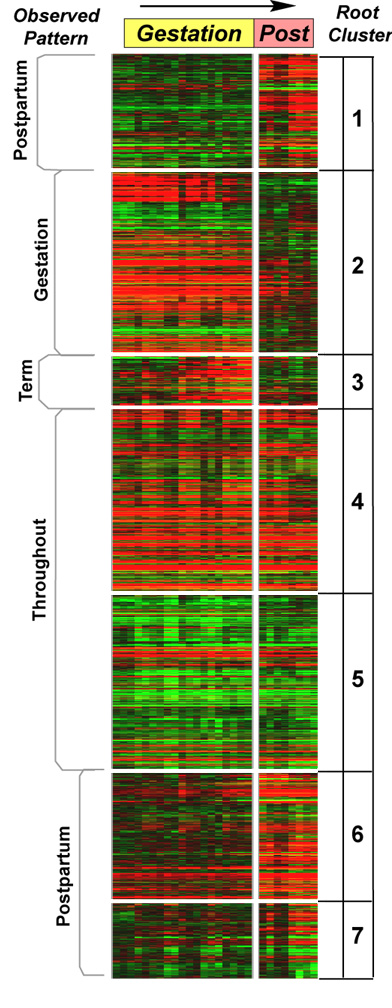

Supplement: Additional data file 2 — A figure showing the HOPACH Absolute Value Pearson Correlation of Myometrial Expression Data [file gb-2005-6-2-r12-s2.jpeg]
