# Supplementary material for: Identifying genetic networks underlying myometrial transition to labor
Source: Genome Biol. 2005 Jan 28;6(2):R12. doi: 10.1186/gb-2005-6-2-r12 (PMC551532; doi:10.1186/gb-2005-6-2-r12)
Supplement: Additional data file 7 — Additional details of methods [file gb-2005-6-2-r12-s7.doc]

**Supplemental Methods:**
**Chromosomal Localization Statistics.** Z scores for genes colocalized in HOPACH and in the genome were calculated by subtracting the expected number of colocalized genes by the observed number of genes, and dividing by the standard deviation of the observed number of genes. The equation used is:

$Z=\frac{(observed-\exp ected)}{std.deviation(observed)}$
or

$$Z=\frac{\left(r-n\frac{R}{N}\right)}{\sqrt{n\left(\frac{R}{N}\right)\left(1-\frac{R}{N}\right)\left(1-\frac{n-1}{N-1}\right)}}$$

where *N* is the total number of genes on that chromosome, *R* is the total number of genes in the HOPACH cluster interval, *n* is the total number of genes in the genomic interval, and *r* is the number of genes colocalized in HOPACH and in the genome. In addition to a Z score, results from this analysis were further filtered by requiring that at least three genes are colocalized and that no more than twice the number of colocalized genes were present in the genomic interval (in the genome).

**MAPPFinder 2.0 Statistics**. To calculate a permuted p value and an adjusted p value for MAPPFinder results, a non-parametric statistic based on 2000 permutations of the data was included in MAPPFinder 2.0. The gene associations were randomized for each sample, generating a distribution of Z scores for each GO term, which are then used to assign p values (the permute P). In addition we use the Westfall-Young adjustment for multiple testing. This adjustment calculates the family-wise error rate for each sample and accounts for the multiple testing. This adjustment gives the adjusted P.

To eliminate redundant GO terms in our MAPPFinder outputs (due to nesting of GO terms and genes), GO terms were exported from MAPPFinder with the path location within the MAPPFinder GO hierarchy. For each branch path of the results tree, a single term was selected (usually highest Z score) to display in the final results. GO terms from distinct branch paths that contain the same genes were also eliminated by manual examination of these pruned results.
